# ﻿Eastern Colombian Páramo *Liodessus* Guignot, 1939 diving beetles are genetically structured, but show signs of hybridization, with description of new species and subspecies (Coleoptera, Dytiscidae)

**DOI:** 10.3897/zookeys.1143.97461

**Published:** 2023-01-31

**Authors:** Michael Balke, Katja Neven, Adrián Villastrigo, Rodulfo Ospina-Torres, Carlos Prieto, Nicolas Gutierrez Rubiano, Ingrid Lotta, Luisa F. Dueñas, Lars Hendrich

**Affiliations:** 1 SNSB-Zoologische Staatssammlung München, Münchhausenstraße 21, D-81247 Munich, Germany Zoologische Staatssammlung München Munich Germany; 2 GeoBioCenter, Ludwig Maximilians University, Munich, Germany Ludwig Maximilians University Munich Germany; 3 Departamento de Biología, Universidad Nacional de Colombia-Sede Bogotá, Bogotá, Colombia Universidad Nacional de Colombia-Sede Bogotá Bogotá Colombia; 4 Departamento de Biología, Universidad del Atlántico, Barranquilla, Colombia Universidad del Atlántico Barranquilla Colombia; 5 Corporación Universitaria Autónoma del Cauca, Popayán, Colombia Corporación Universitaria Autónoma del Cauca Popayán Colombia

**Keywords:** Colombia, Dytiscidae, eastern cordillera, *Liodessus*, new species, new subspecies, Páramo

## Abstract

We studied *Liodessus* diving beetles from six eastern Colombian Páramo areas, as well as from the Altiplano. We discovered a highly characteristic new species, based on male genital morphology, *Liodessussantarosita***sp. nov.**, in the Páramo de Guantiva-Rusia. Specimens from the Altiplano around Bogotá, and the Páramos of Almorzadero, Chingaza, Matarredonda, Rabanal y Rio Bogotá and Sumapaz form one clade of genetically similar populations based on mitochondrial *Cox1* sequence data. The individuals of this clade are sub-structured according to their geographic distribution. The populations differ from each other mainly in terms of body size and coloration and, at most, subtly in their genital morphology. In two cases, we find putative hybrid populations between Altiplano and Páramo areas. We suggest that the different Páramo populations are in an early phase of speciation, and perhaps already genetically isolated in some cases. They are here assigned subspecies status to highlight these ongoing processes pending more comprehensive geographic sampling and use of genomic data. We refer to this clade as the *Liodessusbogotensis* complex, containing *Liodessusb.bogotensis* Guignot, 1953; *Liodessusb.almorzadero***ssp. nov.**; *Liodessusb.chingaza***ssp. nov.**; *Liodessusb.lacunaviridis* Balke et al., 2021, **stat. nov.**; *Liodessusb.matarredonda***ssp. nov.**, and *Liodessusb.sumapaz***ssp. nov.**

## ﻿Introduction

Diving beetles of the genus *Liodessus* Guignot, 1939 (Guignot 1939) belong to the tribe Bidessini and occur in the New World as well as the Afrotropical Region (Biström 1988; Nilsson and Hájek 2022). They are typically smaller than 3 mm and inhabit a variety of mainly lotic habitats. Andean species reach altitudes of nearly 5,000 m, where they are the most abundant aquatic beetles (Balke et al. 2020a, b).

However, diving beetles from the high altitudes of the Puna and Páramo regions have remained poorly studied until recently. Since 2019, as the result of research and training cooperation between our institutions, we regularly provide updates on the high-altitude fauna (e.g. Megna et al. 2019; Balke et al. 2020a, b). It has become apparent that many more new species of *Liodessus* remain to be discovered in the vast Andean highland ecosystems; most of these undiscovered species are likely endemic to one or a few Páramo or Puna areas. To address this integrating morphological as well as molecular evidence, we are building a DNA sequence-based platform for the study of these insects, using the Barcode of Life Data System (BOLD) of the Canadian Centre for DNA Barcoding and the 5' mitochondrial *Cox1* gene fragment (www.boldsystems.org; Ratnasingham and Hebert 2007). The rationale behind this approach was in detail described by Balke et al. (2021b). The public COLLI project on BOLD currently contains 276 sequences for the 5' end of the cytochrome *c* oxidase subunit I for 14 species.

Here, we focus on taxa from the eastern mountain range of Colombia, specifically the Altiplano around Bogotá, and the Páramos of Almorzadero, Chingaza, Guantiva-Rusia, Matarredonda, Rabanal y Rio Bogotá, and Sumapaz.

To date, three *Liodessus* species have been reported from the eastern Colombian branch of the Andes; i.e., *L.bogotensis*, *L.lacunaviridis*, and *L.picinus* (Megna et al. 2019; Balke et al. 2021a, b).

The goals of this publication are (1) to present a new species with highly characteristic male genitalia from the Páramo de Guantiva-Rusia and (2) to reveal a complex of apparently subspecific taxa within *Liodessusbogotensis* Guignot, 1953. The treatment of this complex is based on mitochondrial *Cox1* sequence data as well as male genitalia, where specimens are genetically well structured, with various degrees of supporting morphological features such as genital shape, and body size, shape, and coloration. We here treat them as a complex of subspecies to flag the studied populations, until further genomic evidence and increased geographic sampling help in determining whether these should be considered separate species. Whatever the status of these taxa, this study shows that the Páramo areas studied harbor unique biodiversity worthy of adequate conservation efforts.

## ﻿Materials and methods

### ﻿Study area and map generation

**Figure 1.**
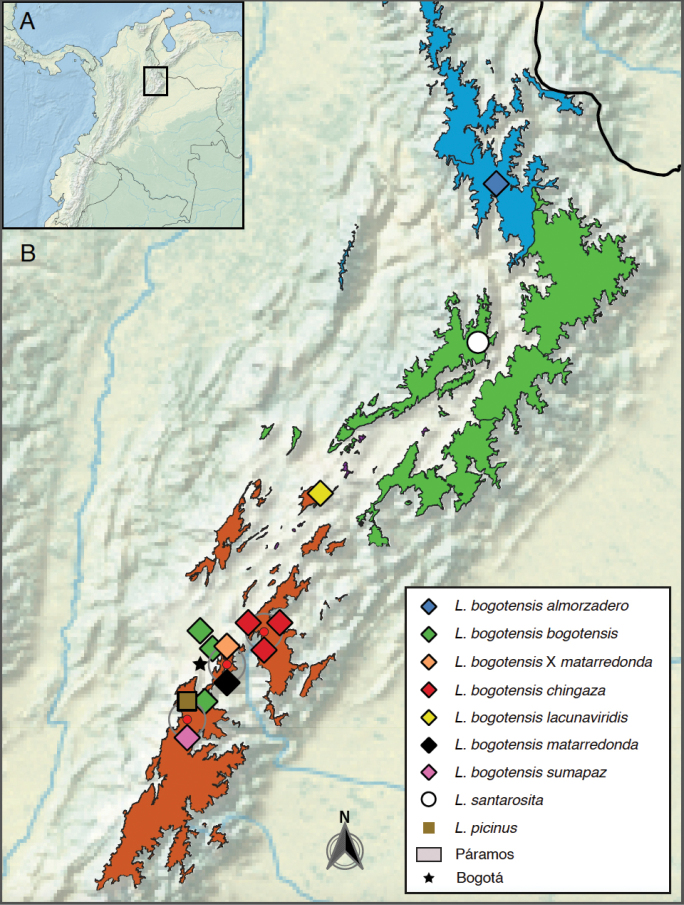
Map of sampling localities **A** overview of northwestern South America showing are of sampling depicted in “B” **B** colored areas represent districts in Páramo: Santanderes (blue), Boyacá (green), and Cundinamarca (brown).

Our sampling includes specimens collected in the Altiplano around Bogotá and the Páramos of Almorzadero, Chingaza, Matarredonda, Rabanal y Río Bogotá, and Sumapaz (Fig. 1). The naming of Páramo follows the “Atlas de páramos de Colombia” (Morales et al. 2007). Our map was generated with QGIS v. 3.16.4 (http://www.qgis.org) using the Natural Earth raster map as a base map and the cartography of the Colombian Páramos (IAVH 2013).

### ﻿Acronyms

**UNAL**Insect collection, Universidad Nacional de Colombia, Bogotá, Colombia;

**ZSM**SNSB-Zoologische Staatssammlung, München, Germany, temporarily stored for further morphological work.

Codes in the format COL_MB_2022_004 in the studied material sections are ZSM locality codes, and refer to the country of origin (**COL** = Colombia), collector who organized the fieldwork (**MB** = Michael Balke), year of collection (2022) and a locality number for the respective collecting event (004).

### ﻿Morphological descriptions and photography

The description of morphological characters follows our previous work on *Liodessus* beetles (Balke et al. 2020b).

Images were taken with a Canon EOS R camera. We used Mitutoyo 10× and 20× ELWD Plan Apo objectives for photographing habitus and genital structures, respectively. These were attached to a Carl Zeiss Jena Sonnar 3.5/135 MC. Illumination was with three LED segments SN-1 from Stonemaster (https://www.stonemaster-onlineshop.de). Image stacks were generated using the Stackmaster macro rail (Stonemaster), and images were assembled with Helicon Focus v. 7.61 on a MacPro 2019 with a Radeon Pro 6800X MPX GPU.

### ﻿DNA analysis

The DNA sequencing and data analysis laboratory protocol follows standard Canadian Centre for DNA Barcoding (CCDB) barcoding procedures (https://ccdb.ca). We delivered tissue samples to CCDB, which were processed and the barcode data (COI-5) uploaded to BOLD systems. We used a simple approach to calculate a neighbor-joining tree (*p*-distances) in Geneious v. 11.0.4. to determine if newly added entries could be assigned to existing species groups. This approach has been proven helpful and in guiding the morphological descriptive process.

### ﻿Diagnostic characters

We aligned the 276 COLLI sequences using the “BOLD > sequence analysis > diagnostic characters” option to detect diagnostic characters mentioned below (Table 1). Data were aligned using the MUSCLE algorithm (Edgar 2004), which resulted in a 658-bp alignment that was inspected using Geneious software.

**Table 1.** Diagnostic characters in the *Cox1* alignment of the COLLI project on BOLD webportal. Above two taxa relative to all other entries in COLLI, and below, within the *L.bogotensis* complex. Numbers above indicate nucleotide position in the 658 bp alignment.

Taxon, Number of sequences	19	22	25	49	58	59	82	88	91	106	127	206	217	223	232	256	286	290	304	316	325
*Liodessussantarosita* (6)	**C**		**T**	**C**	**T**				**A**			**C**		**T**	**A**	**C**	**T**	**A**			
*Liodessusbogotensis* (55)																					
*L.bogotensisalmorzadero* (4)			**G**					**A**													
*L.bogotensisbogotensis* (21)						**T**															
*L.bogotensischingaza* (16)		**A**								**T**	**C**										
*L.bogotensislacunaviridis* (4)							**C**												**A**	**A**	**G**
*L.bogotensismatarredonda* (5)													**G**								
*L.bogotensissumapaz* (5)																					
	**331**	**334**	**337**	**352**	**355**	**358**	**412**	**421**	**445**	**472**	**473**	**490**	**511**	**542**	**547**	**548**	**553**	**586**	**616**	**643**	**655**
*Liodessussantarosita*		**G**	**T**	**A**		**C**			**C**								**G**	**A**		**T**	
*Liodessusbogotensis*					**T**										**C**						
*L.bogotensisalmorzadero*														**T**							
*L.bogotensisbogotensis*																					
*L.bogotensischingaza*										**G**		**C**									**G**
*L.bogotensislacunaviridis*	**G**						**G**	**C**								**T**			**T**		
*L.bogotensismatarredonda*																					
*L.bogotensissumapaz*											**A**		**G**								

### ﻿Haplotype network, fixation index, and genetic distance calculation

**Figure 2.**
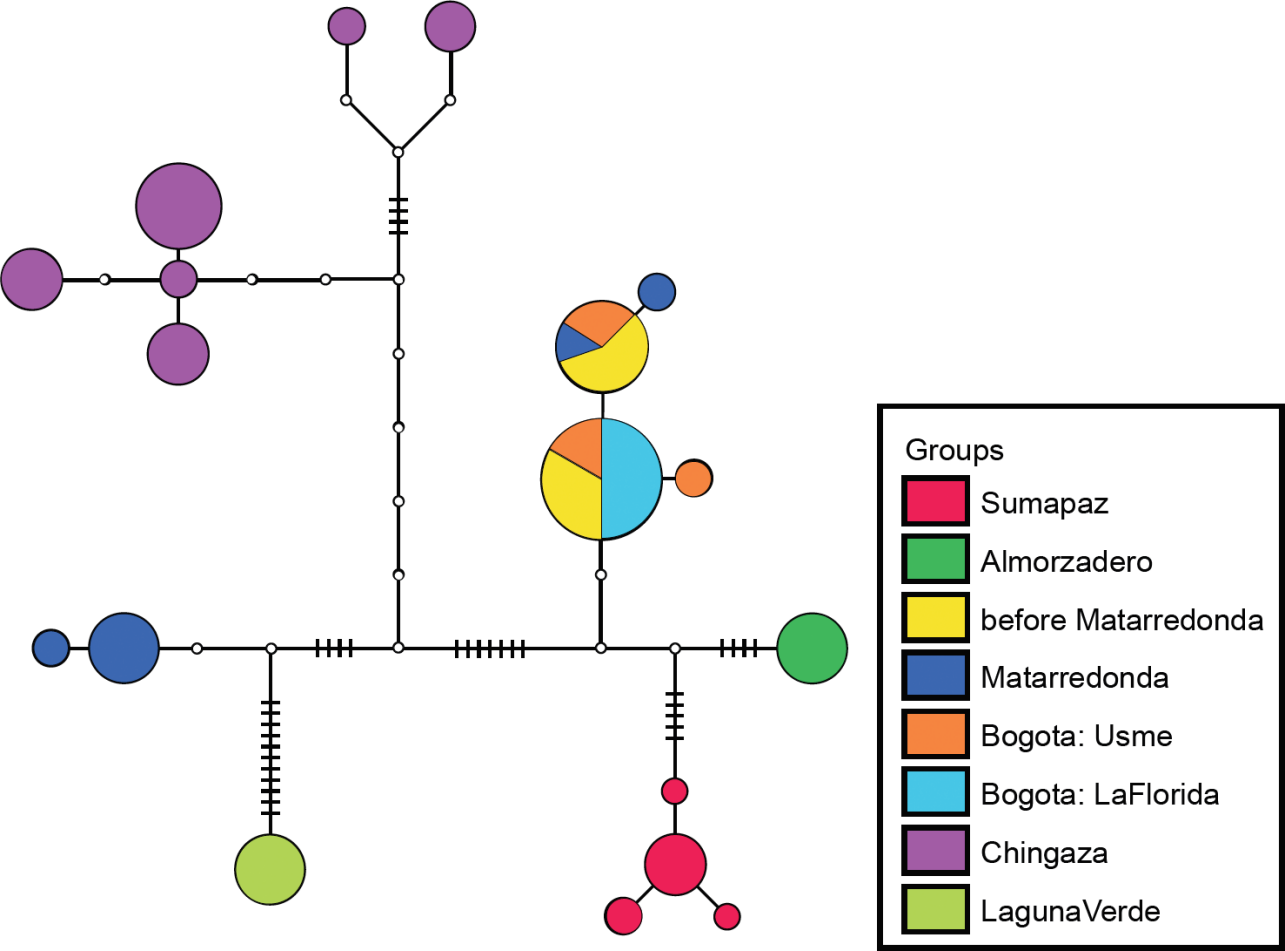
Haplotype network for the *Liodessusbogotensis* complex using the partial gene COI-5.

DNA sequences from the *L.bogotensis* complex were aligned and haplotypes were inferred using PHASE (Stephens et al. 2001) for 1,000 iterations, considering a thinning parameter of 5 and a burn-in fraction of 10% iterations. A haplotype network (Fig. 2) was reconstructed with TCS software (Clement et al. 2000) using a broad-connection threshold to attain a fully connected network. Visualization of the haplotype network was conducted with tcsBu (Múrias dos Santos et al. 2016) using collecting localities as groups for enhanced visualization.

**Figure 3.**
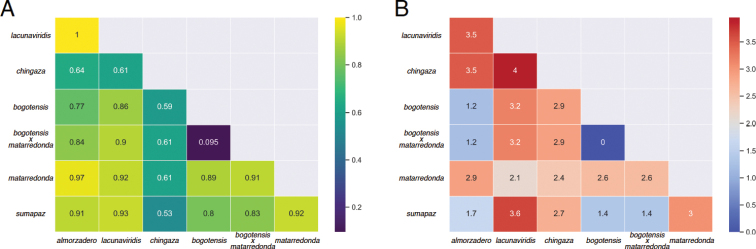
Heatmap of pairwise comparison of *F_ST_* values (**A**) and Minimum genetic divergence (**B**) among the clusters as defined by the haplotype network.

We also computed the fixation index (*F_ST_*) as a measure of population differentiation. *F_ST_* values ranges from 0 to 1 and are an indication whether populations freely interbreed (panmixis, value of 0) or if gene flow is absent and populations are genetically isolated (value of 1). For this, we used DNAsp v. 6 (Rozas et al. 2017), defining the clusters found in the haplotype network as sets of sequences (Fig. 3). Moreover, minimum genetic divergence between populations were calculated using Geneious v. 11.04.

## ﻿Results and discussion

### ﻿A new *Liodessus* species from Páramo de Guantiva-Rusia

#### ﻿ Liodessus santarosita sp. nov.

Taxon classificationAnimaliaColeopteraDytiscidae

C7096A8C-799C-5150-B134-89BC9291E178

https://zoobank.org/B86A86A2-282B-4475-B833-3543606E52DC

Fig. 4A–E

##### Type locality.

Santa Rosita, Páramo de Guantiva-Rusia, Boyaca, Colombia.

***Holotype***: Colombia • ♂; Boyaca, Santa Rosita, El Parador de Gallina; 3,200 m alt.; 7.v.2022; 6.1563, -72.7681; Gutierres, Ospina, & Balke leg.; COL_MB_2022_003; UNAL. ***Paratypes***: Colombia • 165 specimens; same data as holotype; UNAL, ZSM. ZSM specimen imaging number for holotype: ZSM-COL-00127.

##### Description of holotype.

**Figure 4.**
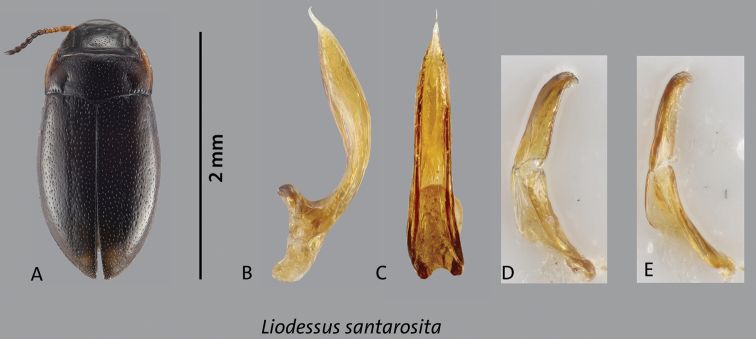
*Liodessussantarosita* sp. nov. **A** habitus **B** median lobe, lateral view **C** median lobe, ventral view **D, E** parameres lateral view. Scale bar: 2 mm.

Habitus with slight discontinuity between pronotum and elytra (Fig. 4A); pronotum widest before base (Fig. 4A). Total length 2.1 mm; length without head 1.8 mm; maximum width 1.0 mm.

***Color*.** Very dark brown to blackish dorsally and ventrally; lighter on lateral pronotum, and bases of meso- and metatibia (Fig. 4A).

***Surface sculpture*.** Head with a few setiferous punctures in front of a distinct occipital line; distinct microreticulation present except on middle of head between eyes (Fig. 4A); posterior to occipital line with distinct microreticulation and a few punctures. Pronotum and elytron shiny, with moderately dense and coarse setiferous punctation (Fig. 4A).

***Structures*.** Head with distinct occipital line, with rounded clypeus. Antenna stout. Pronotum with distinct lateral bead and distinct, long, deep basal striae (Fig. 4A). Elytron with short basal striae.

***Genitalia*.** Median lobe of aedeagus with bulbous main body in lateral view, strongly narrowing at tip and with delicate downwards bent hook; gradually narrowing towards a very narrow, needle-like tip in ventral view (Fig. 4B, C); parameres simple of the “Bidessini” type, 2-segmented (Fig. 4D, E).

##### Variation.

Total length 2.1–2.3 mm (*N* = 20); length without head 1.8–2.1 mm; maximum width 1.0–1.1 mm. In a few specimens, the elytral plicae are fairly obsolete.

***Metathoracic wings*** short, 2/3 of elytral length, venation visible (in one dissected male paratype).

**Female.** External morphology as in male.

##### Etymology.

After the village of Santa Rosita, near the type locality. The word “santarosita” is a noun in the nominative singular standing in apposition.

##### Identification notes.

This species differs from all other *Liodessus* by the needle-like apical part of the median lobe of the aedeagus. In the COLLI sequence database, the species has 19 diagnostic characters different from the other Andean species of the genus (Table 1).

##### Distribution.

Only known from the type locality in the Páramo de Guantiva-Rusia (Fig. 1).

##### Habitat.

Exposed, densely vegetated peatland swamp.

### ﻿The *Liodessusbogotensis* complex

**Figure 5.**
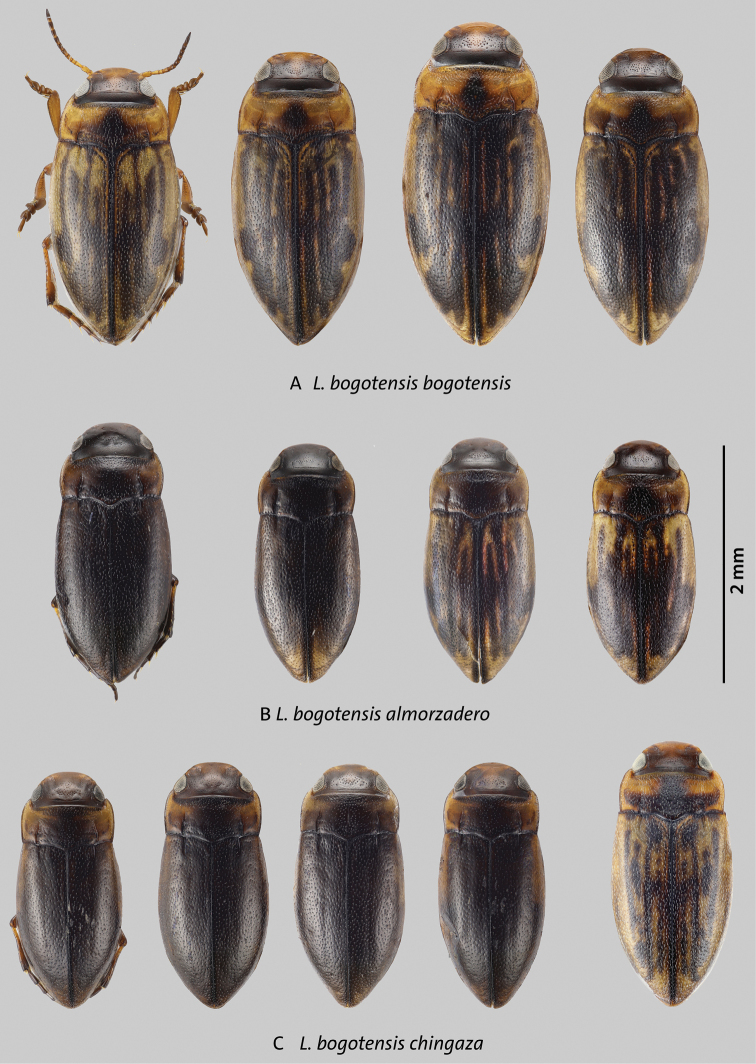
Variation in size and dorsal coloration of subspecies of *Liodessusbogotensis***A***Liodessusb.bogotensis***B***Liodessusb.almorzadero***C***Liodessusb.chingaza*. Scale bar: 2 mm.

**Figure 6.**
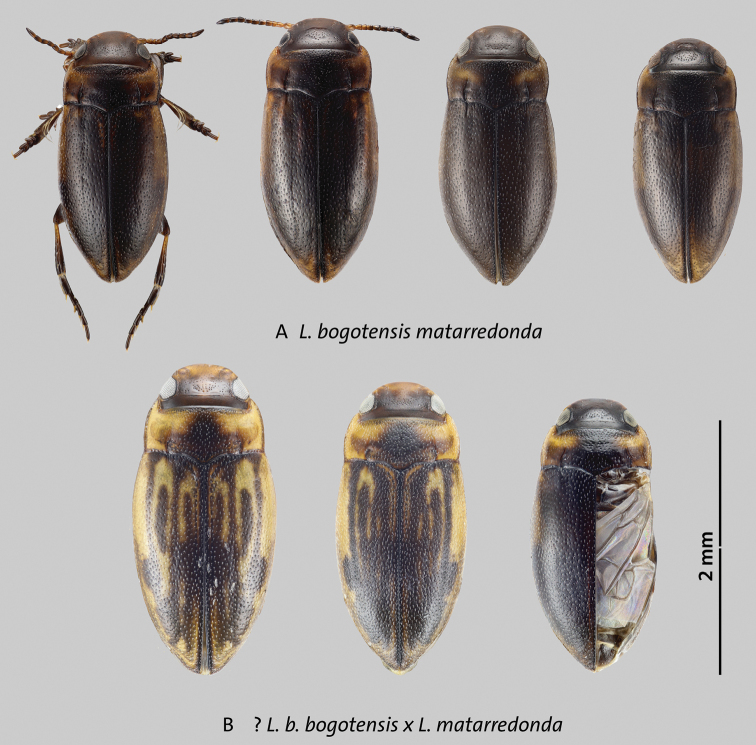
Variation in size and dorsal coloration of subspecies and a hybrid of *Liodessusbogotensis***A***Liodessusb.matarredonda***B** hybrid specimens of *L.b.bogotensis* and *L.b.matarredonda*. Scale bar: 2 mm.

**Figure 7.**
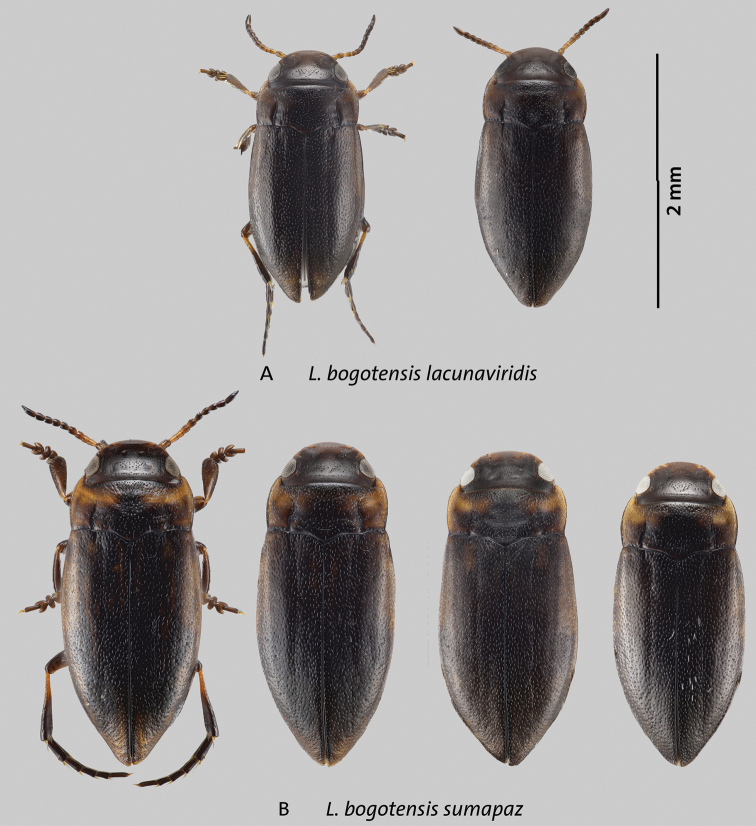
Variation in size and dorsal coloration of subspecies of *Liodessusbogotensis***A***Liodessusb.lacunaviridis***B***Liodessusb.sumapaz*. Scale bar: 2 mm.

**Figure 8.**
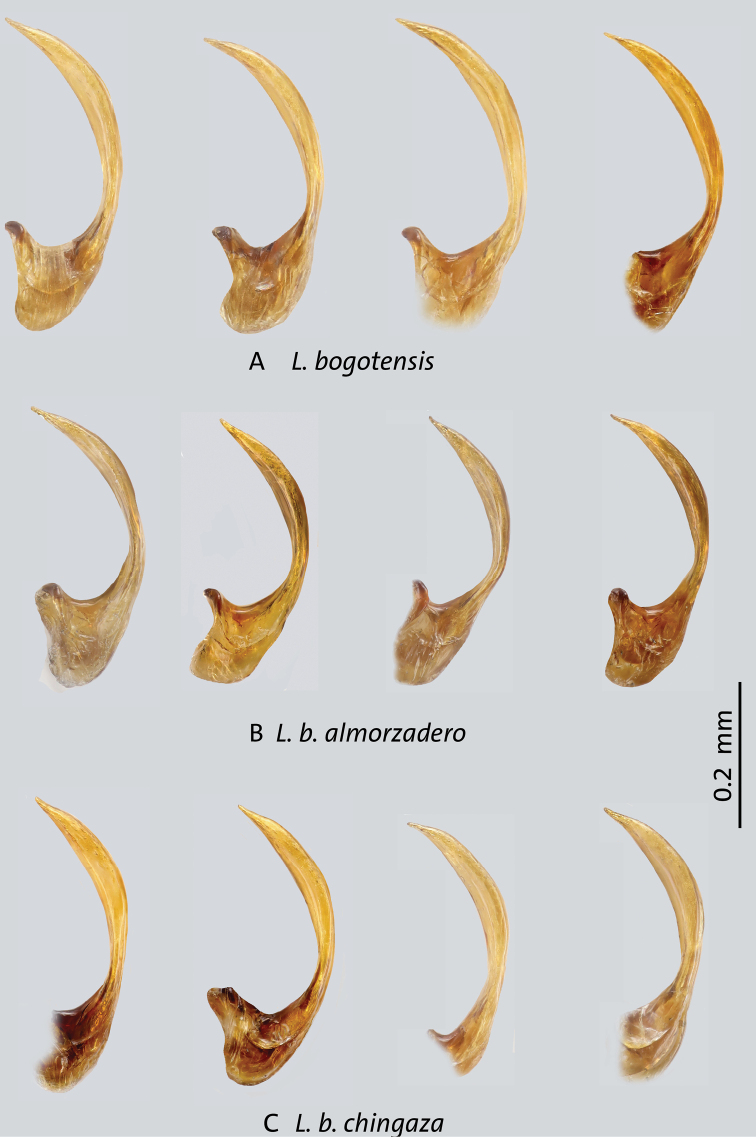
Median lobes in lateral view of *Liodessus* species **A***L.bogotensisbogotensis***B***L.b.almorzadero***C***L.b.chingaza*. Scale bar: 0.2 mm.

**Figure 9.**
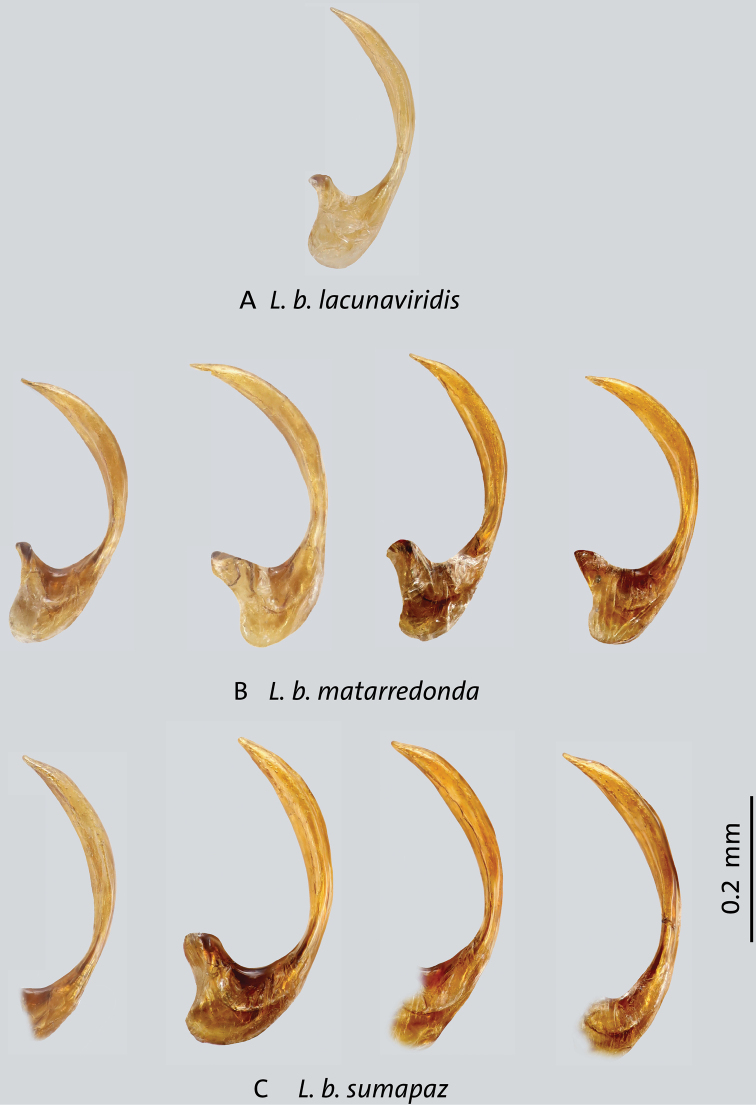
Median lobes in lateral view of *Liodessus* subspecies **A***L.bogotensislacunaviridis***B***L.b.matarredonda***C***L.b.sumapaz*. Scale bar: 0.2 mm.

**Figure 10.**
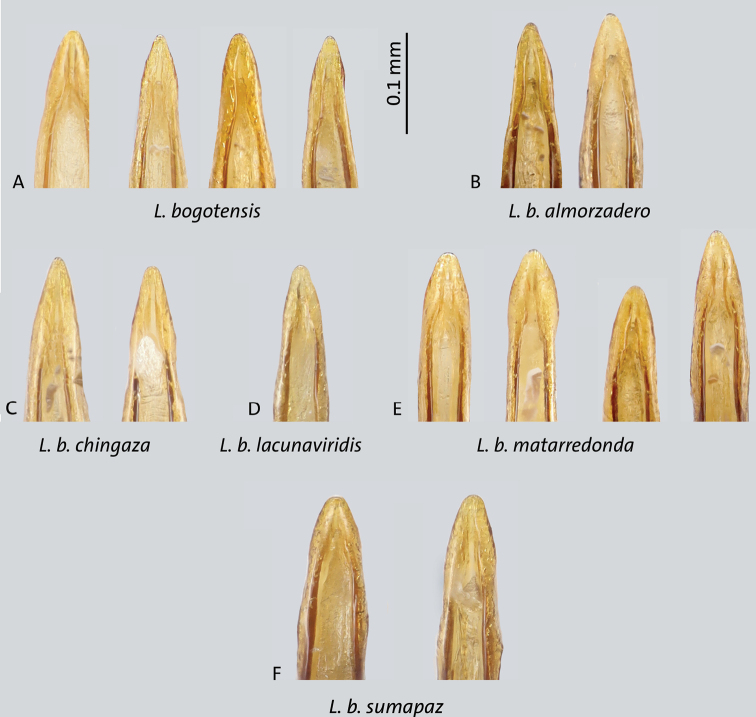
Tips of median lobes in ventral view of *Liodessus* subspecies **A***L.bogotensisbogotensis***B***L.bogotensisalmorzadero***C***L.bogotensischingaza***D***L.b.lacunaviridis***E***L.b.matarredonda***F***L.b.sumapaz*. Scale bar: 0.1 mm.

**Identification notes.** Beetles of this complex are characterized by the following combination of characters: *Liodessus* from the eastern cordillera of Colombia; occipital line usually present, although faint in some specimens; coloration overall dark to bright due to more extensive yellowish or orange dorsal markings (Figs 5–7); median lobe of aedeagus forming a simple curve in lateral and ventral views (Figs 8–10).

In the COLLI sequence database, *L.bogotensis* has two diagnostic characters different from the other Andean species of the genus (Table 1).

**Phylogeographic evidence.** We detected a marked phylogeographic structure among subpopulations of this complex (Figs 2, 3). Six haplotype clusters were recovered, representing five areas in the Páramo as well as the Altiplano around Bogotá up to an altitude of 3,100 m.

Some specimens exhibit intermediate morphological characteristics between the subspecies *L.b.bogotensis* and *L.b.matarredonda* and may be hybrids. They were clustered together with *L.b.bogotensis*. The low *F_ST_* value (*F_ST_* = 0.095) suggests gene flow between the *L.b.bogotensis* and *L.b.matarredonda* populations.

The fixation index, on the other hand, revealed relatively higher population differentiation (*F_ST_* > 0.7) among all other clusters except *Liodessusb.chingaza* (*F_ST_* < 0.7), which might suggest enhanced connectivity of *Liodessusb.chingaza* with other subspecies. However, the genetic divergence of *Liodessusb.chingaza* from the other subspecies is moderate to high compared to the other clusters (more than 2.4% in all cases), which may indicate an ongoing speciation process.

Minimum genetic distances between populations (Fig. 3B) show no divergence between the putative L.b.bogotensis×matarredonda hybrid and *L.b.bogotensis*. Most other subpopulations diverge from each other by 1.2–4.0%, with *L.b.lacunaviridis* the most divergent subpopulation (2.1–4.0%), which is in accordance with its greater genetic isolation (Fig. 1). *Cox1* divergence above 2% is widely assumed to be a rough indicator for the presence of distinct species, from a simple DNA-sequencing perspective (Hebert et al. 2003). However, lineage idiosyncrasies might always blur this picture (Hendrich et al. 2010).

Here, we suggest that ongoing speciation or very young species might be present in *Liodessus* from the eastern cordillera of Colombia. Making this distinction clearly requires greater sampling over the geographic area and the addition of nuclear markers. This study again underpins the high potential and possible pitfalls of using genetic data for taxonomy on the one hand, and the need for morphological examination on the other hand (Meier et al. 2022).

#### ﻿ Liodessus bogotensis bogotensis

Taxon classificationAnimaliaColeopteraDytiscidae

Guignot, 1953

8687BF25-D923-5414-86B0-345255CC1AE0

Figs 5A , 8A , 10A

Liodessus bogotensis Guignot, 1953: 111; Pederzani 2001: 298; Nilsson and Hajek 2022: 122; Megna et al. 2019: 103.

##### Type locality.

Colombia, “Bogota”.

##### Material studied.

Colombia • 10 specimens; Bogota, Usme; 3,100 m alt.; 17.iv.2017; 4.379, -74.12; Megna & Stiven leg.; UNAL, ZSM • 10 specimens; Cundinamarca, Humedal La Florida, 2,400 m alt.; 22.xi.2018; 4.729, -74.143; Ospina, Balke & Megna leg.; COL_MB_2018_12; UNAL, ZSM • 25 specimens; Bogota DC, UNAL campus; 2,500 m alt.; 10.v.2022; 4.6409, -74.0819; Gutierres & Balke leg.; COL_MB_2022_004; UNAL, ZSM.

##### Identification notes.

This subspecies was redescribed by Megna et al. (2019). This is a slightly larger subspecies with a total length of 2.1–2.3 mm. The dorsal color is generally of lighter appearance; the head is chestnut colored to dark orange, the pronotum is yellow with darker marking in the middle, and the elytron is yellow or orange with darker bands or vice versa (Fig. 5A). The median lobe is slender and simply curved in lateral view, with some variation in the degree of tapering at the apex. The median lobe simply narrows towards apex in ventral view (Figs 8A, 10A).

**Figure 11.**
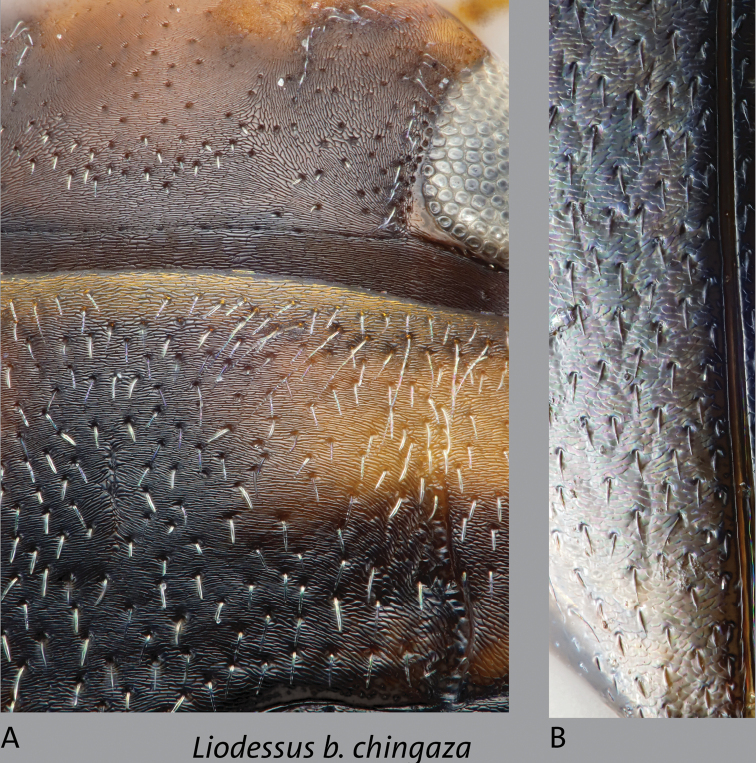
*Liodessusbogotensischingaza* females showing detail of surface reticulation **A** head and pronotum of dull specimen **B** elytral tip of shinier specimen. Not to scale.

An occipital line is present, but sometimes it is faint. Females are sometimes with the entire dorsal surface with strong, dense microreticulation so that they appear dull (mesh-like as in Fig. 11A) or more frequently with the entire dorsal surface with faint, dense microreticulation and appearing shinier. Metathoracic wings are large.

In the COLLI sequence database, only analyzing *L.bogotensis* data, the subspecies has one diagnostic character different from the other subspecies (Table 1).

##### Variation.

Measurements (*N* = 20). Total length 2.1–2.3 mm (mean 2.18); length without head 1.8–2.0 mm (mean 1.89); maximum width 0.9–1.0 mm (mean 0.96).

##### Distribution.

Known from the Altiplano around Bogotá, from 2,400 to 3,100 m, but most likely with wider distribution at moderate altitudes (Fig. 1). See below for possible higher elevation occurrence and hybridization in the Matarredonda area.

##### Habitat.

Exposed, shallow, stagnant densely vegetated water bodies. Collected from swampy areas with thick mats of grass, black mud, and foul water.

#### ﻿ Liodessus bogotensis almorzadero ssp. nov.

Taxon classificationAnimaliaColeopteraDytiscidae

DF583360-AB55-548C-953C-F851A9DB6D1D

https://zoobank.org/40C31742-954E-4152-873B-C8C71923EFC6

Figs 5B , 8B , 10B

##### Type locality.

Colombia, Santander, Páramo del Almorzadero.

***Holotype***: Colombia • ♂; Santander, Páramo del Almorzadero; 3,400 m alt.; 31.xii.2020; 6.9410, -72.6775; Prieto leg.; COL_CPr_2020_001; UNAL. ***Paratypes***: Colombia • 26 specimens; same data as holotype; UNAL, ZSM. ZSM specimen imaging number of holotype: ZSM-COL-00103.

##### Description of holotype.

Habitus narrowly elongate-oval, with slight discontinuity between pronotum and elytra (Fig. 5B); pronotum widest before base (Fig. 5B). Total length 2.0 mm; length without head 1.8 mm; maximum width 0.9 mm.

***Color*.** Very dark brown to blackish dorsally and ventrally; lighter on anterior head, lateral pronotum, and bases of meso- and metatibia (Fig. 5B).

***Surface sculpture*.** Head with a few setiferous punctures in front of a distinct occipital line; fine yet distinct microreticulation present on the entire head. Pronotum and elytron shiny, with moderately dense, coarse, setiferous punctation (Fig. 5B).

***Structures*.** Head with distinct occipital line, with rounded clypeus. Antenna stout. Pronotum with distinct lateral bead and distinct, long, deep basal striae (Fig. 5B). Elytron with distinct, short basal striae.

***Genitalia*.** Median lobe of aedeagus typical of the *L.bogotensis* complex, with slight variation in curvature among individuals in lateral view (Figs 8B, 10B); parameres simple, of the “Bidessini” type, 2-segmented.

##### Variation.

***Measurements*** (*N* = 20). Total length 2.1–2.3 mm (mean 2.18); length without head 1.8–2.0 mm (mean 1.89); maximum width 0.9–1.0 mm (mean 0.96).

***Occipital line*** in a few specimens interrupted, presence still evident.

***Color*.** Usually darkly colored, but a few specimens with paler band-like markings on elytra.

***Microreticulation on head*** sometimes hardly visible on anterior part or frons.

Female variation: see below.

***Metathoracic wings*.** Short, 2/3 of elytral length (in one dissected male paratype).

**Female.** Elytron with microreticulation on about the posterior 1/2 to 2/3.

##### Etymology.

Named after the type locality, Páramo del Almorzadero. The word “almorzadero” is a noun in the nominative singular standing in apposition.

##### Identification notes.

This subspecies is reliably identified only on the basis of the collecting locality and *Cox1* data. In the COLLI sequence database, the subspecies has three diagnostic characters different from the other subspecies (Table 1).

##### Distribution.

Only known from the Páramo del Almorzadero (Fig. 1).

##### Habitat.

Exposed, shallow, densely vegetated, stagnant water bodies.

#### ﻿ Liodessus bogotensis chingaza ssp. nov.

Taxon classificationAnimaliaColeopteraDytiscidae

7093F586-C5F5-5EF8-BB60-94B738AFBDD4

https://zoobank.org/7C652A11-2FC1-44F0-88B6-1D91E17FD047

Figs 5C , 8C , 10C , 11A, B

##### Type locality.

Colombia, Cundinamarca, Páramo de Chingaza.

***Holotype***: Colombia • ♂; Cundinamarca, PN Chingaza; 3,330 m alt.; 14.xi.2018; 4.747, -73.856; Ospina, Balke & Megna leg.; COL_MB_2018_05; UNAL. ***Paratypes***: Colombia • 112 specimens; same data as holotype; UNAL, ZSM • 87 specimens; Cundinamarca, PN Chingaza; 3,500 m alt.; 14.xi.2018; 4.718, -73.821; Ospina, Balke & Megna leg.; COL_MB_2018_06; UNAL, ZSM • 81 specimens; Cundinamarca, PN Chingaza; 3,700 m alt. (3,500 m alt. on label); 15.xi.2018; 4.706, -73.804; Ospina, Balke & Megna leg.; COL_MB_2018_07; UNAL, ZSM. ZSM specimen imaging number for the holoytpe: ZSM-COL-00118.

##### Description of holotype.

Habitus narrowly elongate-oval, with slight discontinuity between pronotum and elytra (Fig. 5C); pronotum widest before base (Fig. 5C). Total length 2.0 mm; length without head 1.8 mm; maximum width 0.9 mm.

***Color*.** Very dark brown to blackish dorsally and ventrally; lighter on anterior head, lateral pronotum, and bases of meso- and metatibia (Fig. 5C).

***Surface sculpture*.** Head with a few setiferous punctures in front of a distinct occipital line, distinct microreticulation present except on middle of head between the eyes (Fig. 5C); posterior to occipital line with distinct microreticulation and a few punctures. Pronotum and elytron shiny, with moderately dense, coarse, setiferous punctation (Fig. 5C).

***Structures*.** Head with distinct occipital line, with rounded clypeus. Antenna stout. Pronotum with distinct lateral bead and distinct, long, deep basal striae (Fig. 5C).

***Genitalia*.** Median lobe of aedeagus typical of the *L.bogotensis* complex, with slight variation in lateral view in curvature among individuals (Figs 8C, 10C); parameres simple, of the “Bidessini” type, 2-segmented.

##### Variation.

***Measurements*** (*N* = 20). Loc. COL_MB_2018_05: total length 2.1–2.3 mm (mean 2.17); length without head 1.8–2.0 mm (mean 1.85); maximum width 0.9–1.0 mm (mean 0.96).

Loc. COL_MB_2018_06: total length: 2.3–2.5 mm (mean 2.39); length without head 1.9–2.2 mm (mean 2.07); maximum width 1.0–1.2 mm (mean 1.08).

***Occipital line*.** This line can be visible, or more or less obsolete, or even absent.

***Color*.** Usually darkly colored but a few specimens with paler band-like markings on elytra.

Female variation: see below.

***Metathoracic wings*.** Polymorphic. Short, 2/3 of elytral length; venation visible in five dissected specimens from COL_MB_2018_05 and COL_MB_2018_07. The wings are longer and larger in five dissected specimens from COL_MB_2018_06.

**Female.** External morphology as in male, with the dorsal surface shiny except for microreticulation on the head; dorsal sculpturation varies in females, with microreticulation on the posterior 1/3 (more or less) of elytron so surface appears slightly less shiny than the males (Fig. 11B), or entire dorsal surface with stronger, dense microreticulation and surface appears dull (Fig. 11A). Dull females were mostly encountered at locality COL_MB_2018_06.

##### Etymology.

Named after the type locality, Chingaza National Park. The word “chingaza” is a noun in the nominative singular standing in apposition.

##### Identification notes.

This subspecies is reliably only identified on the basis of the collecting locality and *Cox1* data. In the COLLI sequence database, this subspecies has six diagnostic characters different from the other subspecies (Table 1).

##### Distribution.

Only known from the Chingaza National Park, Colombia (Fig. 1).

##### Habitat.

Exposed, shallow, densely vegetated, stagnant water bodies.

##### Notes.

Specimens from COL_MB_2018_06 are slightly larger than those from the two other localities in Chingaza. Their wings are slightly longer, the coloration is brighter in some specimens (Fig. 5C, right), and the proportion of dull to shiny females appears to be greater. This makes these specimens somewhat intermediate between *Liodessusb.bogotensis* (Fig. 5A) and *Liodessusb.chingaza*, yet they share the *Liodessusb.chingaza* haplotype (Fig. 2, left purple branch). Specimens with a slightly deviating haplotype (top purple branch) were from COL_MB_2018_07 and agree morphologically with *Liodessusb.chingaza* from COL_MB_2018_05.

#### ﻿ Liodessus bogotensis lacunaviridis

Taxon classificationAnimaliaColeopteraDytiscidae

Balke, Ospina-Torres, Megna & Hendrich, 2021 stat. nov.

9D06E0AA-11E7-5F12-98F0-526DF952CC1B

Figs 7A , 9A , 10D

Liodessus lacunaviridis Balke et al., 2021a: 8.

##### Type locality.

Colombia, Boyacá, Páramo Rabanal y Rio Bogotá, Laguna Verde.

***Holotype***: Colombia • ♂; Boyacá, Laguna Verde; 3,300 m alt.; 28.xi.2017; 5.4093, -73.5484; Ospina & Balke leg.; COL_MB_2017_03; UNAL.

##### Notes and diagnosis.

*Liodessuslacunaviridis* was described by Balke et al. (2021a). This is a smaller taxon in the genus *Liodessus* (body length 1.8–2.0 mm; Fig. 7A). It is morphologically and genetically (*Cox1* data) different from *L.bogotensis*, and we suggested that this as a new species. Although this might still be warranted, in the context of the current study, at least some morphologically and genetically distinct Páramo populations of the *L.bogotensis* complex in the Eastern Cordillera seem to be in contact and hybridize with the generally lower-elevation *L.bogotensis*. We suggest here to treat all these populations as subspecies pending availability of genomic data and greater sampling for more areas.

The median lobe is slender and simply curved in lateral view, and simply narrowing towards the apex in ventral view (Fig. 9A). In dorsal view, this is a dark beetle (Fig. 7A). The occipital line is either distinct or poorly defined, consisting of serial punctures. The metathoracic wings are short, less than 2/3 of the elytral length.

##### Variation.

***Measurements***: total length 1.8–2.0 mm; length without head 1.6–1.8 mm; maximum width 0.9–1.0 mm.

##### Identification notes.

This subspecies is only reliably identified on the basis of the collecting locality and *Cox1* data. In the COLLI sequence database this subspecies has nine diagnostic characters different from the other subspecies (Table 1).

##### Distribution.

Known from the Páramo del Rabanal y Rio Bogotá, where it has been collected near Laguna Verde (Fig. 1).

##### Habitat.

Exposed, shallow, densely vegetated, stagnant bodies of water.

#### ﻿ Liodessus bogotensis matarredonda ssp. nov.

Taxon classificationAnimaliaColeopteraDytiscidae

DF47C319-B4BD-5E73-9EF4-51860EC0BF73

https://zoobank.org/ABB5619B-5148-4FFF-8FB7-C7C319325C0A

Figs 6A , 9B , 10E

##### Type locality.

Colombia, Cundinamarca, Páramo de Matarredonda.

***Holotype***: Colombia • ♂; Cundinamarca, Matarredonda park; 3,400 m alt.; 20.xi.2018; 4.560, -74.013; Ospina, Balke & Megna leg.; COL_MB_2018_10; UNAL. ***Paratypes***: Colombia • 126 specimens; same data as holotype; UNAL, ZSM. ZSM specimen imaging number for holotype: ZSM-COL-00084.

##### Description of holotype.

Habitus narrowly elongate-oval, with a slight discontinuity between pronotum and elytra (Fig. 6A); pronotum widest before base (Fig. 6A). Total length 2.0 mm; length without head 1.8 mm; maximum width 0.9 mm.

***Color*.** Very dark brown to blackish dorsally and ventrally; lighter on anterior head, lateral pronotum, and bases of meso- and metatibia; elytron with slightly paler band like markings obvious under dorsal lighting under dissecting scope. (Fig. 6A).

***Surface sculpture*.** Head with a few setiferous punctures in front of a faint occipital line, distinct microreticulation present except on middle of head between the eyes (Fig. 6A); posterior to occipital line with distinct microreticulation and few punctures. Pronotum and elytron shiny, with moderately dense, coarse, setiferous punctation (Fig. 6A).

***Structures*.** Head with faint occipital line, with rounded clypeus. Antenna stout. Pronotum with distinct lateral bead and distinct, long, deep basal striae (Fig. 6A).

***Genitalia*.** Median lobe of aedeagus typical of the *L.bogotensis* complex, with slight variation in curvature among individuals, somewhat more bulbous before tip; tip in ventral view more parallel-sided (Figs 9B, 10E); parameres simple of the “Bidessini” type, 2-segmented.

##### Variation.

***Measurements*** (*N* = 20). Total length: 1.8–2.1 mm (mean 2.02); length without head 1.7–1.9 mm (mean 1.77); maximum width 0.9–1.0 mm (mean 0.91).

***Occipital line*.** Faint and ill-defined to almost absent (only a few serial punctures visible instead).

***Color*.** Usually darkly colored but a few specimens with paler, band-like markings on elytra.

Female variation: see below.

***Metathoracic wings*.** Short, 2/3 or elytral length; venation visible in five dissected specimens from COL_MB_2018_10.

**Female.** External morphology as in male, with the dorsal surface shiny except for microreticulation on the head; dorsal sculpturation varies, either with microreticulation on the posterior ~1/3 of elytron so the surface appears slightly less shiny, or with microreticulation stronger over the entire dorsal surface so the surface appears dull (mesh-like, as in Fig. 11A).

##### Etymology.

Named after the type locality, Páramo de Matarredonda. The word “matarredonda” is a noun in the nominative singular standing in apposition.

##### Identification notes.

The median lobe of the aedeagus is slightly more parallel-sided in ventral view than in the other subspecies (Fig. 10 E). This subspecies is only reliably identified on the basis of the collecting locality and *Cox1* data. In the COLLI sequence database this subspecies has one diagnostic character different from the other subspecies (Table 1).

##### Distribution.

Only known from the Páramo de Matarredonda but see below (Fig. 1).

##### Habitat.

Exposed, shallow, densely vegetated, stagnant bodies of water.

##### Note.

Specimens from a locality at 3,300 m en route to the Páramo de Matarredonda from Bogota city are morphologically intermediate between *L.bogotensismatarredonda* and *L.b.bogotensis* (Fig. 6B), and they most likely belong to a hybrid population. Their haplotype is that of *L.b.bogotensis* (Fig. 2).

#### ﻿? Liodessus bogotensis bogotensis × Liodessus bogotensis matarredonda

Taxon classificationAnimaliaColeopteraDytiscidae

23ABCECD-D5A2-5424-B3C6-7BEE669E1274

Fig. 6B

##### Material studied.

Colombia • 67 specimens; Cundinamarca, pools by the road from Bogotá before Matarredonda park; 3,300 m alt.; 20.xi.2018; 4.564, -74.008; Ospina, Balke & Megna leg.; COL_MB_2018_09; UNAL, ZSM.

##### Notes.

This form is intermediate between two subspecies, in terms of body size and coloration (Fig. 6B). Females are mostly completely microreticulation dorsally as in *L.b.bogotensis*, but in some the head and pronotum are shiny, and the elytra have microreticulation only apically. The metathoracic wings are large, as in *L.b.bogotensis*. Some specimens determined by body size and dorsal coloration would be assigned to *L.b.matarredonda*, but are fully winged (Fig. 6B).

##### Measurements

**(N = 20).** Total length 2.2–2.5 mm (mean 2.36); length without head 1.9–2.2 mm (mean 2.02); maximum width 1.1–1.2 mm (mean 1.07).

##### Distribution.

Below Páramo de Matarredonda (Fig. 1).

#### ﻿ Liodessus bogotensis sumapaz ssp. nov.

Taxon classificationAnimaliaColeopteraDytiscidae

EAA73E83-4FD8-5F08-8905-17E23CFFAD5E

https://zoobank.org/53689F1E-E38C-46C6-A54A-627B2D5831D6

Figs 7B , 9C , 10F

##### Type locality.

Colombia, Bogotá D.C., Páramo de Sumapaz.

***Holotype***: Colombia • ♂; Bogotá D.C., PN Sumapaz; 3,500 m alt.; 13.xi.2018, 4.290, -74.207; Ospina, Balke & Megna leg.; COL_MB_2018_04; UNAL. ***Paratypes***: Colombia • 409 specimens; same data as holotype; UNAL, ZSM. ZSM specimen imaging number for holotype: ZSM-COL-00096.

##### Description of holotype.

A larger representative of the genus. Habitus narrowly elongate-oval, with slight discontinuity between pronotum and elytra (Fig. 7B); pronotum widest before base (Fig. 7B). Total length 2.5 mm; length without head 2.1 mm; maximum width 1.1 mm.

***Color*.** Very dark brown to blackish dorsally and ventrally; lighter on anterior head, lateral pronotum, and bases of meso- and metatibia; elytron with slightly paler, band-like markings obvious under dissecting scope (Fig. 7B).

***Surface sculpture*.** Head with a few setiferous punctures in front of a faint occipital line; distinct microreticulation only present around the eyes (Fig. 7B); posterior to occipital line with distinct microreticulation and few punctures. Pronotum and elytron shiny, with moderately dense, coarse, setiferous punctation (Fig. 7B).

***Structures*.** Head with a rather faint occipital line in which there is series of punctures, with rounded clypeus. Antenna stout. Pronotum with distinct lateral bead and distinct, long, deep basal striae (Fig. 7B).

***Genitalia*.** Median lobe of aedeagus typical of the *L.bogotensis* complex, with slight variation in curvature among individuals; tip in ventral view subtly broader than in the other subspecies of the complex (Figs 9C, 10F); parameres simple, of the “Bidessini” type, 2-segmented.

##### Variation.

***Measurements*** (*N* = 20). Total length 2.4–2.6 mm (mean 2.53); length without head 2.1–2.3 mm (mean 2.19); maximum width 1.0–1.2 mm (mean 1.1).

***Occipital line*.** Faint but well visible, or more or less obsolete (a few punctures visible instead).

***Color*.** Usually darkly colored but a few specimens with paler, band-like markings on elytra.

Female variation: see below.

***Metathoracic wings*.** Short, 2/3 or elytral length, venation visible in five dissected specimens from COL_MB_2018_10.

**Female.** External morphology as in male, with the dorsal surface shiny except for microreticulation on the head; dorsal sculpture varies, with microreticulation on the posterior ~1/3 of elytron so that the surface appears slightly less shiny, or entire dorsal surface with stronger, dense microreticulation so that the surface appears dull (e.g. Fig. 7B, second specimen from right).

##### Etymology.

Named after the type locality, Páramo de Sumapaz. The word “sumapaz” is a noun in the nominative singular standing in apposition.

##### Identification notes.

This subspecies is reliably only identified on the basis of the collecting locality and *Cox1* data. In the COLLI sequence database this subspecies has two diagnostic characters different from the other subspecies (Table 1).

##### Distribution.

Only known from the Páramo de Sumapaz (Fig. 1).

##### Habitat.

Exposed, shallow, densely vegetated, stagnant water bodies.

## Supplementary Material

XML Treatment for Liodessus santarosita

XML Treatment for Liodessus bogotensis bogotensis

XML Treatment for Liodessus bogotensis almorzadero

XML Treatment for Liodessus bogotensis chingaza

XML Treatment for Liodessus bogotensis lacunaviridis

XML Treatment for Liodessus bogotensis matarredonda

XML Treatment for Liodessus bogotensis bogotensis × Liodessus bogotensis matarredonda

XML Treatment for Liodessus bogotensis sumapaz
